# Designing Preclinical Studies in Germline Gene Editing: Scientific and Ethical Aspects

**DOI:** 10.1007/s11673-019-09947-9

**Published:** 2019-11-21

**Authors:** Anders Nordgren

**Affiliations:** Centre for Applied Ethics, Linköping University, 58183 Linköping, Sweden

**Keywords:** Animal experimentation, Embryo research, Gene editing, Germline, Preclinical research

## Abstract

Human germline gene editing is often debated in hypothetical terms: if it were safe and efficient, on what further conditions would it then be ethically acceptable? This paper takes another course. The key question is: how can scientists reduce uncertainty about safety and efficiency to a level that may justify initiation of first-time clinical trials? The only way to proceed is by well-designed preclinical studies. However, what kinds of investigation should preclinical studies include and what specific conditions should they satisfy in order to be considered well-designed? It is argued that multispecies and multigenerational animal studies are needed as well as human embryo editing without implantation. In order to be possible to translate to first-time clinical trials, animal studies need to satisfy strict conditions of validity. Moreover, embryo studies intended for translation to first-time clinical trials need to correspond to the animal studies in experimental design (with exception of implantation). Only in this way can uncertainty about risk for harm (safety) and prospect of benefit (efficiency) in first-time clinical trials be reduced to a modest level. If uncertainty is not reduced to such a level, first-time clinical trials in germline gene editing should not be initiated.

## Introduction

Gene editing has emerged as a promising approach in many areas of bioscience, especially with the development of the CRISPR/Cas9 technology. Gene editing in humans may in principle be carried out on somatic cells as well as on the germline (Nuffield Council on Bioethics 2016; National Academies of Sciences, Engineering, and Medicine 2017). While the ethical debate on somatic gene editing has been rather modest, the debate on germline editing has been heated and in many respects similar to the debate twenty years ago on germline gene therapy. The main difference between the CRISPR technology and older technologies, such as ZFNs and TALENs, is that it is cheaper, easier to use, and requires much shorter time for experiments. This makes it a “disruptor” technology and the issue of clinical application more pressing (Mulvihill et al. 2017).

Human germline gene editing is often debated in hypothetical terms: if it could be made safe and efficient, on what further conditions would it then be ethically acceptable? This paper takes another course. The key question is: how can scientists reduce uncertainty about safety and efficiency to a level that may justify initiation of first-time clinical trials? The only way to proceed is by well-designed preclinical studies. However, what kinds of investigation should preclinical studies include and what specific conditions should they satisfy in order to be considered well-designed? The aim of this paper is to provide tentative answers to these questions.

The importance of preclinical research on germline gene editing has recently been emphasized by the European Society of Human Genetics and the European Society of Human Reproduction and Embryology:

Both for scientific and moral reasons, as a precondition for any potential clinical applications of GLGE [germline gene editing], adequate pre-clinical research on GLGE is necessary. Pre-clinical research, involving both animal and human embryo research, is an important element of the moral framework for the introduction of new, experimental, reproductive technologies generally. Given the specific sensitivity of GLGE, such research would have to take place under ongoing monitoring and societal oversight. Pre-clinical GLGE research would involve investigation of the safety (e.g. possible off-target effects or epigenetic effects) and effectiveness of gene editing in view of possible future applications of GLGE in gametes, zygotes or preimplantation embryos. Such research is important in order to identify and eliminate, or at least reduce, avoidable risks for any future children thus conceived (de Wert, Pennings, et al. 2018, 3).

We notice that these European organisations stress the importance of both animal studies and human embryo studies. However, in the paper they do not specify how these preclinical studies are to be carried out. This is actually quite symptomatic for the present discussion. Surprisingly little has been published about how to design preclinical studies on germline gene editing. Animal research, and sometimes human embryo research, is mentioned in general terms but nothing is stated about the more precise conditions for the research. In the present paper I intend to discuss these conditions in more detail. I will stress that this preclinical research should be well-designed and explain what this would entail.

## Benefit, Risk, and Uncertainty

Preclinical research on germline gene editing would be justified only if germline gene editing has substantial potential clinical benefits and only if its potential clinical benefit outweighs its potential clinical risks. However, potential benefits and risks should be distinguished from actual benefits and risks, and these are uncertain in first-time human trials on genuinely novel interventions such as germline gene editing. Before we turn to this problem and how to handle it, let us have a brief look at potential clinical benefits and risks of germline gene editing as presented in the literature. It should be noted that I focus only on potential benefits and risks of clinical applications, not on potential benefits and risks of enhancement applications.

### Potential Clinical Benefits

The potential clinical benefits of germline gene editing by CRISPR can be indicated by comparing this technology with preimplantation genetic diagnosis (PGD) and somatic gene editing.

It is often stated that germline gene editing would be unnecessary from a clinical perspective because PGD followed by embryo selection in the context of in vitro fertilization (IVF) is preferable in most clinical cases (Lander 2015). However, proponents argue that there might still be cases in which germline gene editing would be beneficial and that this should not be underestimated. First, in some cases PGD is not an option. For example, if one of the prospective parents is homozygous for a dominant disorder, all off-spring will be affected. This is very rare but the benefit should not be ignored, according to proponents. Second, life-threatening diseases such as Huntington’s and Tay-Sachs may be eliminated for good in a family line. Clinicians do not have to carry out PGD over and over again. Proponents argue that if we accept PGD, we should also accept this once for all elimination. Third, it will hardly be practically possible to prevent polygenic disorders such as diabetes, heart disease, schizophrenia, and some types of cancer by PGD, because it would require too many embryos. Gene editing opens up the possibility of making multiple modifications in a single embryo. Proponents admit that this is highly speculative but argue that if it were to work it would be extremely beneficial (Savulescu et al. 2015; Gyngell, Douglas, and Savulescu 2017).

The relative potential merits of germline gene editing compared to somatic gene editing have also been pointed out. The European Society of Human Genetics and the European Society of Human Reproduction and Embryology argue that while somatic gene editing can be very useful in many clinical cases, germline gene editing has certain advantages. One advantage is that it can be more efficient than somatic gene editing in prevention of multiorgan disorders. Another advantage is its potential multigenerational preventive effects (de Wert, Heindryckx, et al. 2018).

### Potential Clinical Risks

In the debate, potential clinical risks with germline gene editing have been stressed by both critics and proponents. Critics argue that gene editing technologies “could have unpredictable effects on future generations,” making “it dangerous and ethically unacceptable” (Lanphier et al. 2015, 410). Also those who are positive to germline gene editing admit that gene editing “could cause” harmful effects (Savulescu et al. 2015, 477). At the same time, however, proponents want to put the risk for harm into perspective by comparing it with other emerging technologies such as information and communication technologies. Although these technologies “could be catastrophic (for example, through cyberterrorism), this does not mean on balance they should be banned. Their expected benefits outweigh their expected harms” (Savulescu et al. 2015, 476–477). So, they argue that the prospect of benefit of these other technologies on balance outweighs their risk for harm and that the same can be expected concerning germline gene editing.

The clinical risks of germline gene editing are of various types. The most commonly stressed risk is off-target mutations. Such unintended mutations may lead to development of cancer or other diseases (Baltimore et al. 2015; Gyngell, Douglas, and Savulescu 2017). Mulvihill and colleagues—including The International Human Genome Organisation (HUGO) Committee of Ethics, Law, and Society (CELS)—describe the “scientific unknowns about human germ line mutagenesis” as a “growing” issue (Mulvihill et al. 2017, 22–23). Moreover, the European Society of Human Genetics and the European Society of Human Reproduction and Embryology stress that gene editing may also have epigenetic effects (without specifying what this could mean more precisely; de Wert, Heindryckx, et al. 2018; de Wert, Pennings, et al. 2018). Another type of risk is unintended effects of on-target mutations (Baltimore et al. 2015). A mutation that is beneficial may still be harmful due to pleiotropic effects. A DNA segment regulating one particular gene may also be involved in regulating another gene. Editing this DNA segment may have unknown systemic effects. Reducing the risk for one disorder may increase the risk of having another (Gyngell, Douglas, and Savulescu 2017; Guttinger 2018; de Wert, Heindryckx, et al. 2018; de Wert, Pennings, et al. 2018).

To be sure, there is substantial disagreement on the potential clinical benefits and risks of germline gene editing. However, in this paper I assume that on balance the potential clinical benefits of germline gene editing may outweigh its potential clinical risks to a degree sufficient to justify preclinical studies on such editing.

### Uncertainty about Risk and Benefit

So far I have talked about potential risk. This should be distinguished from actual risk. The problem is that when we talk about risk in first-time human trials on germline gene editing, we do not know what the actual risks are. We do not have any numerical values. In “decision-making under risk,” decision-makers have sufficient information to assign probabilities to alternative outcomes. This should be distinguished from “decision-making under uncertainty” where this is not the case.

The distinction between decision-making under risk and decision-making under uncertainty was first made by Knight (1921). In recent discussions on emerging technologies several commentators have stressed the importance of this distinction (e.g. Elliott and Dickson 2011; Hansson 2013; cf. Genske and Engel-Glatter who in addition talk about “decision-making under ignorance” (2016)). With this in mind, it seems more adequate to describe decision-making concerning initiation of first-time clinical trials on germline gene editing as decision-making under uncertainty rather than decision-making under risk. If a particular germline gene editing procedure becomes clinical practice after thorough clinical trials, then decision-making under uncertainty would be replaced by decision-making under risk. Clinicians would then have a good enough basis of knowledge to assign probabilities to alternative outcomes.

Let me add that decision-making under uncertainty may concern not only risk for harm but also prospect of benefit. We saw above, that according to proponents, germline gene editing has certain potential clinical benefits. It might be useful for phenotypic prevention, that is, helping individuals to avoid serious health problems. However, in first-time clinical trials we do not know what the actual benefits will be. We cannot assign probabilities to alternative outcomes. The prospect of benefit is uncertain.

### The Goal of Preclinical Research: Reduction of Uncertainty

Now, what is the proper response to uncertainty about risk for harm (safety) and prospect of benefit (efficiency) in first-time clinical trials on germline gene editing? It seems to be measures that reduce the translational distance between preclinical investigations, for example animal studies, and first-time clinical trials. This idea of reduction—but not elimination—of translational distance has been clearly expressed by Kimmelman in his book on first-in-human trials on somatic gene transfer (2010). Kimmelman states:

In considering the ethics of trial initiation, the dissimilarity between preclinical experiments and clinical trials might be likened to a kind of distance in inference. The core thesis proposed … is that translational trials should never exceed a modest translational distance; to do otherwise threatens the primary objective of the study–scientific utility–as well as the subject’s welfare (118)

We see here that Kimmelman uses a metaphor of distance in order to describe the inference from preclinical experiments to first-in-human clinical trials and proposes that the translation should never exceed a “modest” distance. This proposal is very much in line with the well-known recommendation by Orkin and Motulsky in their 1995 report to the U.S. National Institutes of Health (NIH). They stressed the necessity of focusing more on preclinical research before initiating first-time clinical trials in somatic gene therapy (Orkin and Motulsky 1995).

So, the goal of preclinical research is reduction of uncertainty about risk for harm and prospect of benefit in first-time clinical trials. This uncertainty should be reduced to a modest level, that is, the translation from preclinical studies to first-time clinical trials should never exceed a modest distance (the conditions for modest translational distance are presented further below). In the quotation we see that Kimmelman presents two reasons for aiming at this goal: scientific utility and the welfare of subjects. Scientific utility is essential, and this requires that preclinical studies are well-designed. If these studies are not well-designed, they cannot be relied upon in translation to first-time clinical trials. This would be unsatisfying from a strictly scientific point of view, but also from an ethical point of view, since this may threaten the welfare of human subjects participating in first-time clinical trials. The risk for harm of study participants should be as limited as possible, even if it cannot be eliminated completely. In first-time clinical trials, risk can never be zero. Results from animal experiments are never completely reliable in clinical application to humans because there might be effects that are specific to humans. However, the risk that remains after well-designed preclinical studies must be balanced against the potential benefit. Only if this potential benefit is very high, could it be justified to continue to first-time clinical studies despite some remaining uncertainty about risk.

## Reduction of Uncertainty by Well-Designed Animal Studies

### A Four-Part Framework

A key component of preclinical research is animal experimentation. Kimmelman suggests a four-part framework for reduction of uncertainty in somatic gene transfer by animal experimentation (Kimmelman 2010, 117–128). Based on a systematic review of guidelines for animal experimentation, Henderson and Kimmelman later refined this framework (Henderson et al. 2013). The four conditions of their framework are as follows.

#### Internal validity

Internal validity is the ability to make inferences concerning causal relationships based on experimental results. This is to be ensured by, for example, randomization and blinding. Many animal studies actually fail in this regard.

#### Construct validity

There needs to be a correspondence of experimental design between preclinical and clinical trials. Animal models should match the manifestation of the human disease, and experiments should involve methods and objectives that resemble those planned for future clinical trials. Clinical trials in fact often include endpoints and other experimental features that differ from those of animal studies, and this diminishes the relevance of animal studies.

#### External validity

External validity concerns whether the causal relationships hold under varied conditions. This can be ensured by, for example, replication in several animal species and testing in different laboratories. If the causal relationships hold in several animal species that are similar to humans in relevant respects, scientists may have at least some reason to assume that they will hold also in humans. In this way external validity of animal experiments is relevant to first-time clinical trials in humans.

#### Credibility

Presentation of supporting evidence should have credibility. Optimism bias and publication bias need to be avoided as well as conflicts of interests (Henderson et al. 2013; Kimmelman 2010, 117–128; see also Baylis and McLeod 2017).

Preclinical studies should be designed in such a way that all four conditions are satisfied. If these four conditions are satisfied, then the translational distance between preclinical studies and first-time clinical trials is modest (Kimmelman 2010, 119). If the translational distance is modest, then uncertainty about risk for harm and prospect of benefit is reduced to a modest level.

The relevance of this framework for gene editing has been clearly shown by Baylis and McLeod in their critical evaluation of the preclinical evidence presented to the Recombinant DNA Advisory Committee (RAC) at NIH in support of the first-in-human Phase 1 CRISPR gene editing cancer trial (Baylis and McLeod 2017). In their evaluation of this somatic gene editing proposal, they systematically apply the four-part framework with several important results. Let me give a few examples. Regarding internal validity, Baylis and McLeod point out that the sample size was “surprisingly small” (seventeen mice) and that only five mice were treated with the CRISPR method. Moreover, there was no randomization or blinding. Construct validity was compromised because the scientists introduced a human lung cancer cell line into the animal model, while the prospective patients would have other types of cancer (melanoma, synovial sarcoma, or multiple myeloma). Moreover, the anatomical location of the cancer differed from the likely location in the patients. Concerning external validity they criticize that a single (small) animal species was used and that the experiments were conducted at only one laboratory. Finally, they questioned the credibility because of indications of optimism bias (Baylis and McLeod 2017). Baylis and McLeod arrived at the following conclusion:

We conclude that the one preclinical study in mice used to justify the first-in-human Phase 1 CRISPR gene editing cancer trial in the United States does not satisfy the ethical requirement of scientific validity. Moreover, the translational distance between the preclinical study and the proposed clinical trial is unnecessarily wide–the quality of preclinical evidence is seriously deficient (310).

So, their conclusion was rather negative. The proposed clinical study on somatic gene editing was lacking in many respects. It was not based on good enough preclinical studies.

I suggest that the four-part framework is relevant also to first-time clinical trials on germline gene editing. The lesson to be learned from the analysis by Baylis and McLeod is that preclinical research on germline gene editing also needs to strictly satisfy the four conditions. Only by reduction of the translational distance from wide to modest by well-designed animal studies, could initiation of first-time clinical trials on germline gene editing be ethically acceptable.

Moreover, Kimmelman’s reasons for aiming at reduction of uncertainty in first-time clinical trials by well-designed preclinical animal studies—scientific utility and welfare of subjects (see above)—are relevant also in the context of first-time clinical trials on germline gene editing. In this case the subjects are the implanted embryos, fetuses, and born human beings. Uncertainty about their welfare should be reduced to a modest level. However, we need to include also the welfare of future generations. In an indirect sense, the welfare of future generations is at stake in any clinical trial, but in germline gene editing it is relevant also in a direct sense, because the specific genetic effects of the editing in a particular first-time clinical trial not only exist in the implanted embryo and the born human being but are also inherited by subsequent generations (the born human being’s children, grandchildren, et cetera). An additional benefit of reducing uncertainty by preclinical animal experimentation is that the general knowledge in germline gene editing gained by such experimentation may indirectly contribute also to future animal welfare. However, improving welfare, for example, of domestic animals with specific animal diseases by germline gene editing would require animal experiments in veterinary medicine directly focusing on such animal diseases rather than animal experiments designed to inform human clinical trials focusing on human diseases.

### Further Comments on Preclinical Studies on Germline Gene Editing

In their policy proposal of a regulatory framework for human germline CRISPR modification, Evitt and colleagues suggest two strategies for preclinical research. The first is research on somatic gene editing, the second animal studies (Evitt, Mascharak, and Altman 2015). These two strategies seem reasonable. However, I have three remarks.

First, although continued research on somatic gene editing may provide information relevant also to germline gene editing, it should not be designed for this purpose. Preclinical studies and clinical trials on somatic gene editing should be designed for possible future clinical application of somatic gene editing, not for germline gene editing. Evitt and colleagues are not clear on this point. Moreover, the information obtained by continued research on somatic gene editing is not sufficient for germline gene editing. It is necessary to include also information that is more directly relevant to germline gene editing than the information gained by research on somatic gene editing, namely information about genetic effects in the germline which are also inherited by subsequent generations. Evitt and colleagues are therefore completely right when they suggest animal experimentation on germline gene editing as a supplement. Multispecies and multigenerational animal studies may at least to some extent reduce uncertainty about what may happen during human fetal development and in future human generations.

Second, as pointed out above, in order to truly contribute to reduction of uncertainty regarding germline gene editing, the animal experiments need to satisfy the conditions of the four-part framework. Because of uncertainty about risk for off-target mutations and unintended long-term effects of on-target mutations, the experiments need to be conducted in multiple animal species of increasing complexity as well as in multiple generations. Both these requirements are correctly pointed out by Evitt and colleagues. However, they do not seem aware of the serious scientific problems related to such animal studies. The animal studies need to strictly satisfy all four conditions of the framework.

Third, in addition to animal studies it is also necessary to carry out preclinical experiments in human embryos (de Wert, Pennings, et al. 2018; see further below). But, as noticed by Mertes and Pennings (2015), Evitt and colleagues do not include preclinical embryo studies in their policy proposal, because of, in their view, the relatively high moral status of embryos: “embryos bear an intermediate moral status between nonhuman life and a fetus” (Evitt, Mascharak, and Altman 2015). However, gene editing in embryos may reduce uncertainty about risk for harm and prospect of benefit in a way that is not possible in animal studies, since this germline gene editing in embryos may provide knowledge about possibilities and problems that are specific to germline gene editing in the human species. By this preclinical strategy, the translational distance may come even closer to being modest than would otherwise be possible if scientists only use the strategy of animal studies (and the strategy of research on somatic gene editing). On the other hand, even if embryo research without implantation is necessary, it is not sufficient. It needs to be supplemented by multispecies and multigenerational animal studies. Only by such studies may scientists reduce uncertainty about what might happen in future generations when the technology is applied in first-time clinical studies. Preclinical embryo studies and animal studies are both necessary.

### Objections and Responses

Let us take a brief look at a couple of possible ethical objections to the preclinical strategy of animal experimentation before we turn to a discussion of the preclinical strategy of embryo research.

The strategy of multispecies and multigenerational animal experimentation might be questioned from an animal welfare perspective. Many more or less radical objections have been presented against animal experimentation in general but this paper is not the proper place to discuss these in detail (for an analysis of various views on animal experimentation in general and on research on genetically engineered animals in particular, see Nordgren 2010). However, let us focus on an objection that stresses the importance of balancing expected human benefit and expected animal suffering on a case-by-case basis. According to this objection, multispecies and multigenerational animal experiments in germline gene editing are unacceptable because sentient animals may suffer and do so in several generations, and this suffering is on balance not outweighed by expected human benefit. Animal welfare may be compromised when they are used as disease models or because of off-target mutations or adverse systemic effects. Moreover, multigenerational animal experiments in germline gene editing may be even less acceptable from an animal welfare perspective than single-generational animal experiments in somatic gene editing, because a higher number of animals is involved.

Scientists who want to carry out these experiments may respond to this objection in the following way. First, they could make the fundamental ethical assumption that humans have higher moral status than sentient non-human animals, at least in the weak sense that our moral obligations to humans (for example, to prevent serious disease) are commonly stronger than those to mice, rats, and other experimental animals, although we have some moral obligations also to them, because they are sentient beings. Among these moral obligations to experimental animals are obligations to minimize their suffering and enrich the environment in their cages (Nordgren 2010, 47–83).

Second, scientists could argue that the expected human benefit of their particular animal experiments is greater than assumed in the objection and that abstaining from these animal experiments would be to expose born human beings participating in first-time clinical trials to unacceptable uncertainty about risk for harm.

Third, scientists could stress that they design their studies according to the 3R (replacement, reduction, refinement) approach proposed by Russell and Burch (1959) and alluded to, for example, in the E.U. directive on animal experimentation (Council of the European Communities 1986, Article 7). Regarding replacement, they could argue that it does not seem possible to replace multispecies and multigenerational animal studies with other alternatives—although this would be something to strive for—because in order to answer the scientific question of how to reduce uncertainty about risk and benefit in germline gene editing, whole animals are needed (not merely computer models or cells) and also whole animals in several generations; otherwise, the systemic and long-term effects of germline gene editing cannot be investigated. Moreover, scientists could argue that they design their studies in such a way that no more animals are used than necessary to answer their scientific question (reduction). Finally, they could argue that they refine their studies scientifically by designing them according to the strict conditions of the four-part framework and thus ensuring that animals are not used in low-quality research. They also refine their studies ethically by minimizing animal suffering and providing good housing and care. In sum, designing animal experiments according to the 3Rs will be a way of minimizing harm to animals while still enabling research for human benefit. The 3Rs can also be seen as a compromise between enabling research and maintaining public trust, as a way of respecting value pluralism on the contested issue of animal experimentation by allowing animal experimentation but also restricting it at least to some extent (on the concept of compromise, see further below on embryo research).

Another possible objection to the strategy of multispecies and multigenerational animal experimentation concerns priority-setting. Some critics may argue that it would be more justified to perform preclinical animal studies in somatic gene editing than in germline gene editing. Multigenerational animal studies in germline gene editing would involve a higher number of animals and be carried out over longer experimental periods of time than single-generation studies in somatic gene editing, and therefore be more expensive. Moreover, somatic gene editing seems to be a clinically more promising technology than germline gene editing. There is too much uncertainty and too much disagreement concerning germline gene editing compared to somatic gene editing. With this in mind, it seems more justified to use limited public research resources for reducing uncertainty in somatic gene editing than in germline gene editing, or perhaps even more justified to use the resources for other more urgent types of biomedical research.

To this, scientists could respond that the prospect of human benefit from animal studies in germline gene editing might be greater than assumed in the objection and that it therefore would be justified to allocate substantial public resources to these studies. Because so much is at stake with regard to benefit as well as risk, it is justified to prioritize animal studies on germline gene editing in order to reduce uncertainty. Moreover, even if these animal experiments do not lead to clinical application, the animal experiments could still be valuable enough as basic research to justify giving priority to such experiments. However, if allocation of public resources to animal studies in germline gene editing still cannot be justified, there is also the option of private funding.

## Reduction of Uncertainty by Well-Designed Embryo Studies

### Conditions for Preclinical Gene Editing in Embryos

Well-designed animal experiments are just one step in the preclinical research necessary for reducing uncertainty in translation to first-time clinical trials. As argued above, preclinical studies in human embryos are also necessary.

While human germline gene editing is prohibited in many parts of the world (Isasi, Kleiderman, and Knoppers 2016; Kipling 2016), preclinical research on embryos up to fourteen days is allowed in several countries (for example, the United States, the United Kingdom, Belgium, and Sweden). However, there are differences concerning how embryos are allowed to be obtained. A few countries allow creation of embryos for research purposes. Many more countries allow research on surplus embryos produced in the context of IVF (Mertes and Pennings 2015; Kipling 2016).

A serious problem in germline gene editing in embryos is mosaicism. The problem has been detected in editing in tripronuclear zygotes (Liang et al. 2015; Kang et al. 2016). It is considered particularly serious in editing of surplus embryos, since most of these embryos are beyond the cleavage stage. Mosaicism makes detection of the outcome with PGD inaccurate (Vassena et al. 2016; de Wert, Heindryckx, et al. 2018). In order to avoid mosaicism, alternatives to the use of zygotes and surplus embryos are, for example, application of CRISPR at the time of sperm injection (Ma et al. 2017), use of progenitors of egg and sperm (National Academies of Sciences, Engineering, and Medicine 2017, 85), and use of induced pluripotent stem (iPS) cells (Vassena et al. 2016).

This preclinical embryo research has to satisfy four conditions.

#### No implantation of edited embryos

Preclinical gene editing in embryos should only be conducted in vitro. The edited embryo should not be implanted into the uterus. Implanting edited embryos would be a radical step that should not be taken before uncertainty about risk and benefit has been reduced to a modest level, that is, the translational distance has been reduced from wide to modest. To take a leap directly from animal studies to implantation of edited human embryos would be ethically irresponsible. It would mean that uncertainty about risks are not taken seriously enough. On the other hand, this presupposes that embryos lack moral status or at least full moral status. Reducing uncertainty about risk for harm in future first-time clinical trials is judged to carry more moral weight than discarding embryos after preclinical editing (for objections to this view, see further below).

#### Embryo research in vitro only up to fourteen days

Recently, a debate has arisen about whether or not to revise the fourteen-day rule (Hyun, Wilkerson, and Johnston 2016). The background is that embryos were reported to be kept alive in vitro for twelve to thirteen days after fertilization, while earlier only up to seven days (Deglincerti et al. 2016; Shahbazi et al. 2016). The fourteen-day rule was established as a policy tool—by, for instance, the Warnock commission in the 1980s—to find a balance between enabling research and maintaining public trust (Cavaliere 2017). Day fourteen was considered appropriate, because it signifies the point of individuation at which the primitive streak appears and twinning and fusion is no longer possible (Pera 2017). Those in favour of an extension of the rule by a few days refer to new promising options for research. However, I agree with Cavaliere that the reasons for changing this policy are presently not strong enough. Moreover, any future proposal needs to be based on careful balancing of expected scientific benefit and public views on embryos (Cavaliere 2017).

#### The four-part framework

The conditions for modest translational distance between preclinical animal studies and first-time clinical trials hold also for modest translational distance between preclinical embryo studies and first-time clinical trials. Also, in this case internal validity is vital. The embryo experiment must be designed in such a way that adequate causal inferences can be drawn. In order to satisfy the condition of construct validity, it is essential that exactly the same experimental procedures and methods are used as in the planned first-time clinical trial (with exception of implantation). External validity is to be ensured by replication and testing in different laboratories. Credibility requires that, for example, optimism bias is avoided in presentation of scientific results.

#### Correspondence between animal studies and human embryo studies

The four conditions hold also for modest translational distance between animal studies and embryo studies. Of particular importance is correspondence (construct validity) between the design of the embryo studies and the design of the animal studies (with exception of implantation: in preclinical animal editing animal embryos should be implanted, while in preclinical human embryo editing human embryos should not be implanted). For instance, the CRISPR intervention in an embryo study should target exactly the same disorder—such as the same specific type of cancer—and be carried out in exactly the same manner, whether it is applied at the time of sperm injection or in any other way. However, such correspondence is not necessary at an early stage of research, that is, in basic research on embryo editing or in embryo research aiming merely at establishing proof of concept. It is necessary only in translational research in order to increase the translational value of embryo studies.

### Comments on Recent Examples of Embryo Editing

There are already a few examples of research on gene editing in human embryos. Scientists in China applied CRISPR technology to human embryos in order to modify a gene causing a potentially fatal blood disorder (beta-thalassaemia) but stopped when the technology only worked on half of the embryos and there was a surprisingly high number of off-target mutations (Liang et al. 2015). In another study by Chinese scientists, the genes of non-viable human embryos were modified in order to assess CRISPR technology and the option of making precise genetic modifications (Kang et al. 2016). Scientists from the United States, China, and Korea led by Mitalipov reported correction of a mutation for hypertrophic cardiomyopathy in human embryos. CRISPR/Cas9 was co-injected with sperm, the harmful mutation was cut and replaced with a normal copy of the gene from the mother (Ma et al. 2017; see also a comment in Winblad and Lanner 2017). CRISPR technology was used in the United Kingdom for understanding gene function in the development of human embryos (Fogarty et al. 2017).

These studies satisfy the first two conditions for embryo research on germline gene editing mentioned above. The embryos are not implanted, and the studies are terminated before fourteen days. However, it is not clear to what extent these studies satisfy the last two conditions. The articles seem to report basic research or research aiming at establishing proof of concept. They refer to some animal studies but not to animal studies that specifically correspond to the reported embryo studies. This is acceptable at a preliminary stage but diminishes their translational value for first-time clinical trials. Well-designed multispecies and multigenerational animal studies satisfying the four-part framework are necessary at some point to increase the translational value of embryo research on the editing procedure. Only in this way, can uncertainty about risks and benefits be reduced to a level that may justify initiation of first-time clinical trials.

### Objections and Responses

Preclinical research on germline gene editing in human embryos is rather controversial. Let me briefly respond to a few possible ethical objections to this strategy.

A fundamental objection is that embryo research is ethically unacceptable because it does not respect the full moral status of embryos. Discarding embryos is not consistent with respecting their moral status (Deckers 2005).

Scientists who want to carry out these embryo experiments in germline gene editing may respond as follows. First, they could make the basic ethical assumption that early human embryos lack moral status or at least full moral status and that born human beings have higher moral significance than early embryos. A possible argument for this view could be that born human beings are sentient and can feel pain, while this is not the case for early embryos. Another argument is that early embryos are merely potential human beings lacking moral status or at least full moral status, not actual human beings with full moral status. Both arguments have been disputed (for a critical analysis, see Deckers 2005), but to discuss this fundamental issue is beyond the purpose of this paper.

Second, scientists could argue that to take a leap directly from animal studies to first-time clinical studies (with implantation of the embryo) without taking the intermediate step of embryo studies (without implantation) would be to expose born human beings and future generations to unacceptable uncertainty about risk for harm.

Third, scientists could focus on the political level and argue that a policy allowing germline gene editing research on embryos up to fourteen days is a reasonable compromise that respects the value pluralism in society and at the same time enables research. Substantial moral disagreements exist concerning embryo research. These disagreements seem impossible to eliminate. However, society needs to find ways of handling these disagreements, and compromise appears in this case acceptable from the perspective of political liberalism. Compromise means that each party gives up part of their demand. In this case, those in favour of unrestricted embryo research give up their preference and those against embryo research give up their preference and agree on a compromise, namely the fourteen-day rule. As mentioned above, the 3R approach can in a similar manner be seen as a compromise regarding animal experimentation. The reason for aiming at compromise is that this seems to be the best way to respect value pluralism, and only if value pluralism is respected can scientists obtain public trust (for an analysis of the argument of public trust and the argument of respect for value pluralism in the context of embryo research, see Cavaliere 2017).

Another objection against embryo studies without implantation stresses the processual nature of organisms, including human beings, and the difficulty of evaluating the effects of germline gene editing. Even if uncertainty regarding off-target mutations could be reduced by detection within the range of the fourteen-day rule, this preclinical research would not be sufficient to reduce uncertainty about systemic effects. This would require that the embryo is implanted, that the embryo, fetus, and born human being are monitored over the whole lifespan, and that the effects of editing are assessed also in subsequent generations. However, implanting embryos would not be ethically acceptable, because this would require that the risk right from the start is almost zero, and that is not possible (Guttinger 2018; Lanphier et al. 2015; Comitato Nazionale Per La Bioetica 2017).

This objection should not be taken lightly. However, scientists could argue that it overstates the problem. Multispecies and multigenerational animal studies may play an important role in reducing uncertainty about risk for adverse long-term systemic effects (safety) and prospect of benefit (efficiency) and in this way supplement the reduction of uncertainty resulting from embryo research without implantation. Conversely, embryo studies without implantation may be a useful supplement to multispecies and multigenerational animal studies by detecting at least some problems that are specific to humans compared to experimental animals. Moreover, the goal of preclinical research is not to completely eliminate uncertainty but to reduce it to a modest level. It is possible that there are effects that are specific to humans that cannot be detected in multigenerational animal studies, and it is possible that there are systemic effects that can be detected only after implantation of the human embryo, during fetal development, in the born human being, or in subsequent generations. However, the remaining uncertainty must be balanced against the potential benefit. Only if the potential benefit of a specific first-time clinical trial is very high and the translational distance based on well-designed preclinical studies is modest, might it be justified to initiate this first-time clinical trial. However, if adverse effects are detected in multispecies and multigenerational animal studies or in embryo studies without implantation, this first-time clinical trial should not be initiated.

A third objection is that PGD with embryo selection at least at present is a sufficiently good alternative to germline gene editing (Cavaliere 2018).

However, as pointed out above, there might still be several ways in which germline gene editing could be beneficial and this would justify that preclinical research is carried out. Moreover, even if PGD would be preferable from the perspective of clinical outcome and cost-effectiveness, preclinical research on gene editing in embryos may still yield important basic research results. We might gain a better understanding of fundamental biological processes and gene function in human development (Mertes and Pennings 2015). Specifically, we might learn more about problems related to human germline mutagenesis, the “growing” issue pointed out by Mulvihill and colleagues (see above; Mulvihill et al. 2017, 22–23). However, it is important to make a distinction between allowing embryo research on gene editing and giving priority to it. Even if society allows such research, it might still not find it justified at present to allocate substantial public resources to it. Probably only a few countries would find this preclinical research urgent enough to give it priority. On the other hand, this does not mean that private funding of such research should not be allowed.

A fourth objection is that preclinical research on gene editing in embryos might lead to a slippery slope. Once we start with preclinical studies on germline gene editing without implantation, this would sooner or later lead to clinical studies with implantation (for an analysis of different versions of this type of argument, see Walton 2017).

To this, scientists could respond with Mertes and Pennings that preclinical studies might lead to clinical studies (and that might be good) or they might not (we might see the problems more clearly and abstain from clinical studies) (Mertes and Pennings 2015). The conclusion after a substantial amount of preclinical research might be that germline editing will not be sufficiently safe and efficient and that the clinical advantages will not outweigh the disadvantages. Preclinical research on germline gene editing without implantation is an important step in reducing uncertainty, but not necessarily a step toward genetic modification of future generations.

## Conclusion

In this paper I have investigated an important requirement for initiation of first-time clinical trials in germline gene editing, namely well-designed preclinical studies. I have argued that multispecies and multigenerational animal studies are needed as well as embryo studies without implantation. In order to be translatable to first-time clinical studies, animal studies need to satisfy strict conditions of internal validity, construct validity, external validity, and credibility. Moreover, embryo studies intended for translation to first-time clinical studies need to be closely related to the animal studies. In particular there needs to be a correspondence in experimental design (with exception of implantation). Only in this way can uncertainty about risk for harm (safety) and prospect of benefit (efficiency) in first-time clinical trials be reduced to a modest level. If uncertainty is not reduced to such a level, first-time clinical trials in germline gene editing should not be initiated.
